# A next-generation newborn screening pilot study: NGS on dried blood spots detects causal mutations in patients with inherited metabolic diseases

**DOI:** 10.1038/s41598-017-18038-x

**Published:** 2017-12-15

**Authors:** F. Boemer, C. Fasquelle, S. d’Otreppe, C. Josse, V. Dideberg, K. Segers, V. Guissard, V. Capraro, FG. Debray, V. Bours

**Affiliations:** 1 Biochemical Genetics Lab, Department of Human Genetics, CHU Sart-Tilman, University of Liège, Liège, Belgium; 2 Human Genetics Unit, GIGA, University of Liège, Liège, Belgium; 3 Molecular Genetics Lab, Department of Human Genetics, CHU Sart-Tilman, University of Liège, Liège, Belgium; 4 Molecular Core Facilities, CHU Sart-Tilman, University of Liège, Liège, Belgium; 5 Metabolic Unit, Department of Human Genetics, CHU Sart-Tilman, University of Liège, Liège, Belgium; 6 Department of Human Genetics, CHU Sart-Tilman, University of Liège, Liège, Belgium

## Abstract

The range of applications performed on dried blood spots (DBS) widely broadened during the past decades to now include next-generation sequencing (NGS). Previous publications provided a general overview of NGS capacities on DBS-extracted DNA but did not focus on the identification of specific disorders. We thus aimed to demonstrate that NGS was reliable for detecting pathogenic mutations on genomic material extracted from DBS. Assuming the future implementation of NGS technologies into newborn screening (NBS), we conducted a pilot study on fifteen patients with inherited metabolic disorders. Blood was collected from DBS. Whole-exome sequencing was performed, and sequences were analyzed with a specific focus on genes related to NBS. Results were compared to the known pathogenic mutations previously identified by Sanger sequencing. Causal mutations were readily characterized, and multiple polymorphisms have been identified. According to variant database prediction, an unexplained homozygote pathogenic mutation, unrelated to patient’s disorder, was also found in one sample. While amount and quality of DBS-extracted DNA are adequate to identify causal mutations by NGS, bioinformatics analysis revealed critical drawbacks: coverage fluctuations between regions, difficulties in identifying insertions/deletions, and inconsistent reliability of database-referenced variants. Nevertheless, results of this study lead us to consider future perspectives regarding “next-generation” NBS.

## Introduction

Next-generation sequencing (NGS) has revolutionized the world of molecular diagnosis over the last decade. This technological evolution has allowed for the sequencing of millions of genomes and exomes, and the exponential increase in related publications is proportional to the gradual decline in cost^1^. To date, the methodology has mainly been applied in clinical settings on high-quality DNA samples (whole blood) or on DNA extracted from formalin-fixed, paraffin-embedded tissues^2^, but protocols have not yet been clinically validated on certain challenging materials such as degraded DNA from forensic samples^3^ or dried blood spots (DBS).

Blood collection on filter paper has evolved as a reference procedure for the collection, transport, analysis and storage of biological fluids. For over 50 years, this sampling protocol has been the key to newborn screening programs worldwide. The Clinical Laboratory Standards Institute (CLSI) periodically edits its corresponding guidelines^4^. Currently, the range of applications performed using filter paper has widely broadened and includes, among other, diet follow-up in metabolic disorders (e.g., phenylketonuria)^5^, therapeutic drug monitoring^6^, doping control^7^, viral load measurements^8^ and targeted gene sequencing^9^. Accordingly, the number of PubMed (www.ncbi.nlm.nih.gov/pubmed)-referenced publications associated with “dried blood spots” item is greatly increasing.

Considering the growing interest in DBS testing, it was worth evaluating whether whole-exome sequencing of such material could detect specific inborn errors of metabolism (IEM) identified by biochemical methods and/or Sanger sequencing. Previous generic publications already reported that filter paper could be used for such a purpose^10–12^, but these papers provided a general overview of technological capacities (i.e., coverage, error rate, number of single nucleotide polymorphisms (SNPs)) and did not focus on the identification of specific disorders or mutations.

Assuming future implementation of NGS technologies into newborn screening (NBS), we conducted a preliminary study sequencing whole exomes on DBS specifically issued from patients with well-established IEM. We interpreted our data with a specific focus on genes related to NBS programs, thus aiming to demonstrate that DBS is an appropriate material for future NBS programs relying on high-throughput sequencing technologies.

## Results

### DNA Extraction

Genomic material was extracted from five blood spots (3.1 mm) simultaneously. DNA integrity was assessed using the KAPA hgDNA Quantification and QC^®^ kit. The amounts of isolated DNA fluctuated between 62 and 248 ng. Q-ratios were close to 1 for all samples, suggesting that the quality of the extracted DNA was reliable.

### Sequencing

A focus was initially set on identifying disorders included in the official newborn screening program of the French Community of Belgium (FWB). Accordingly, Table 1 synthesizes the different diseases and their corresponding mutations for the 15 tested patients.

**Table 1:** Disorders analyzed by exome sequencing using DBS.

**Patient ID**	**Disorder**	**Gene**	**Mutation(s) Allele 1**	**Mutation(s) Allele 2**	**Genomic coordinates Allele 1**	**Genomic coordinates Allele 2**	**Transcript**	**Comment**
DBS-1	PKU	*PAH*	c.482T > C	c.1222 > T	chr12:103260401	chr12:103234271	ENST00000553106	
DBS-2	PKU	*PAH*	c.473G > A	c.842C > T	chr12:103260410	chr12:103246593	ENST00000553106	
DBS-3	MCAD	*ACADM*	c.985A > G	c.985A > G	chr1:76226846	chr1:76226846	ENST00000420607	
DBS-4	Propionic Acidemia	*PCCB*	c.990_991 insT	c.1252G > A	chr3:136035800	chr3:136046050	ENST00000469217	
DBS-5	Methylmalonic Aciduria	*MMAB*	c.556C > T	c.563 − 577dup	chr12:109998873	chr12:109998851	ENST00000545712	
DBS-6	Tyrosinemia type I	*FAH*	c.554–1G > T	c.554 − 1G > T	chr15:80460605	chr15:80460605	ENST00000407106	
DBS-7	Glutaric Aciduria type I	*GCDH*	c.371G > A	c.1204C > T	chr19:13004333	chr19:13008638	ENST00000222214	
DBS-8	3-MCC^a^	*MCCC2*	c.1423G > A	c.1535A > C	chr5:70945945	chr5:70948542	ENST00000340941	
DBS-9	Propionic Acidemia	*PCCB*	c.997delA	c.763G > A	chr3:136035813	chr3:136012706	ENST00000469217	
DBS-10	Homocystinuria	*CBS*	c.429C > G	c.833T > C	chr21:44486375	chr21:44483184	ENST00000398165	
DBS-11	PKU	*DHPR*	c.661C > T	c.661C > T	chr4:17488828	chr4:17488828	ENST00000281243	
DBS-12	Galactosemia	*GALT*	c.563A > G	c. − 119delGTCA^d^	chr9:34648167	chr9:34646583	ENST00000378842	Allele 2 corresponds to Duarte 2 haplotype
				c.378 − 27G > C		chr9:34647802		
				c.507+62G > A		chr9:34648020		
				c.508 − 24G > A		chr9:34648088		
				c.940A > G		chr9:34649442		
DBS-13	MADD	*ETDFH*	c.293T > A	c.293T > A	chr4:159603464	chr4:159603464	ENST00000511912	
DBS-14	MSUD^b^	?^c^	?^c^	?^c^				
DBS-15	MCAD	*ACADM*	c.985A > G	c.1091T > C	chr1:76226846	chr1:76226952	ENST00000420607	

Patient’s pathogenic mutations were first characterized by Sanger sequencing during diagnostic workup. ^a^Disorder not mandated by the newborn screening program of the French community of Belgium.

^b^Maple syrup urine disease.

^c^Sanger sequencing has not been performed for the MSUD patient.

^d^Mutation not covered by our exome sequencing probes.

The bioinformatics flowchart of whole-exome sequencing (WES) was designed to specifically target the 35 IEM genes involved in the NBS program of the FWB and 74 additional genes involved in disorders included or under discussion for inclusion in different official NBS programs^13–16^. Among these additional disorders, we also considered some specific treatable conditions that cannot be identified with reliable biomarkers but that could benefit from early intervention, such as pyridoxine-dependent epilepsy or serine biosynthesis defects (Table 2).

**Table 2:** Disorders and corresponding genes generally considered by NBS programs.

**IEM currently screened in FWB**	**Genes**	**Disorders considered by different NBS programs or initiatives worldwide**	**Genes**
Phenylketonuria	*PAH*	Cystic Fibrosis	*CFTR*
Phenylketonuria	*PTS*	Congenital Adrenal Hyperplasia	*CYP21A2*
Phenylketonuria	*GCH1*	Biotinidase deficiency	*BTD*
Phenylketonuria	*QDPR*	3-Methylcrotonyl-CoA Carboxylase	*MCCC2*
Phenylketonuria	*PCBD1*	Hemoglobin disorders	*HBB*
MSUD	*DBT*	Hemoglobin disorders	*HBA1*
MSUD	*BCKDHA*	Hemoglobin disorders	*HBA2*
MSUD	*BCKDHB*	G6PD deficiency	*G6PD*
Tyrosinemia	*FAH*	Alpha1-Antitrypsin deficiency	*SERPINA1*
Tyrosinemia	*TAT*	Duchenne-Becker dystrophy	*DMD*
Tyrosinemia	*HPD*	Hurler disease	*IDUA*
Homocystinuria	*CBS*	Hunter disease	*IDS*
Homocystinuria	*MTHFR*	Morquio disease	*GALNS*
Homocystinuria	*MTRR*	Maroteaux-Lamy syndrome	*ARSB*
Homocystinuria	*MTR*	Gaucher disease	*BGBA*
Galactosemia	*GALT*	Niemann–Pick A/B disease	*SMPD1*
Galactosemia	*GALK1*	Pompe disease	*GAA*
Galactosemia	*GALE*	Krabbe disease	*GALC*
Methylmalonic Acidemia	*MUT*	Fabry disease	*GLA*
Methylmalonic Acidemia	*MMACHC*	X-Adrenoleukodystrophy	*ABCD1*
Methylmalonic Acidemia	*MMADHC*	Spinal Muscular Atrophy	*SMN1*
Methylmalonic Acidemia	*LMBRD1*	Cerebral Creatine deficiency syndrome	GATM
Methylmalonic Acidemia	*HCFC1*	Cerebral Creatine deficiency syndrome	GAMT
Methylmalonic Acidemia	*MMAA*	Cerebral Creatine deficiency syndrome	SLC6A8
Methylmalonic Acidemia	*MMAB*	Pyridoxine-Dependent Epilepsy	ALDH7A1
Methylmalonic Acidemia	*TCN2*	Pyridoxine-Dependent Epilepsy	PNPO
Propionic Acidemia	*PCCA*	Serine Biosynthesis defect	PHGDH
Propionic Acidemia	*PCCB*	Serine Biosynthesis defect	PSPH
Glutaric Aciduria type I	*GCDH*	Serine Biosynthesis defect	PSAT1
Isovaleric Acidemia	*IVD*	Severe Combined Immunodeficiency	IL2RG
MCAD	*ACADM*	Severe Combined Immunodeficiency	JAK3
MADD	*ETFDH*	Severe Combined Immunodeficiency	IL7RA
MADD	*ETFA*	Severe Combined Immunodeficiency	IL2RA
MADD	*ETFB*	Severe Combined Immunodeficiency	PTPRC
VLCAD	*ACADVL*	Severe Combined Immunodeficiency	CD3D
		Severe Combined Immunodeficiency	CD3E
		Severe Combined Immunodeficiency	CD3Z
		Severe Combined Immunodeficiency	CORO1A
		Severe Combined Immunodeficiency	RAG1
		Severe Combined Immunodeficiency	RAG2
		Severe Combined Immunodeficiency	DCLRE1C
		Severe Combined Immunodeficiency	PRKDC
		Severe Combined Immunodeficiency	AK2
		Severe Combined Immunodeficiency	ADA
		Severe Combined Immunodeficiency	LIG4
		Severe Combined Immunodeficiency	NHEJ1
		Severe Combined Immunodeficiency	CD3G
		Severe Combined Immunodeficiency	CD8A
		Severe Combined Immunodeficiency	PNP
		Severe Combined Immunodeficiency	RMRP
		Severe Combined Immunodeficiency	ZAP70
		Severe Combined Immunodeficiency	CD40LG
		Severe Combined Immunodeficiency	FOXP3
		Severe Combined Immunodeficiency	IL10RA
		Congenital Hypothyroidism^a^	TSHR
		Congenital Hypothyroidism^a^	THRA
		Congenital Hypothyroidism^a^	THRB
		Congenital Hypothyroidism^a^	FOXE1
		Congenital Hypothyroidism^a^	NKX2–1
		Congenital Hypothyroidism^a^	NKX2-5
		Congenital Hypothyroidism^a^	PAX8
		Congenital Hypothyroidism^a^	SLC26A4
		Congenital Hypothyroidism^a^	FOXI1
		Congenital Hypothyroidism^a^	KAT6B
		Congenital Hypothyroidism^a^	KCNJ10
		Congenital Hypothyroidism^a^	UBR1
		Congenital Hypothyroidism^a^	GNAS
		Congenital Hypothyroidism^a^	SLC16A2
		Congenital Hypothyroidism^a^	TPO
		Congenital Hypothyroidism^a^	SLC5A5
		Congenital Hypothyroidism^a^	DUOX2
		Congenital Hypothyroidism^a^	DUOXA2
		Congenital Hypothyroidism^a^	IYD
		Congenital Hypothyroidism^a^	SECISBP2

A. IEM screened in the FWB. B. Additional conditions involved in different NBS programs, including some specific treatable disorders not identifiable with reliable biomarkers.^a^Molecular etiology of congenital hypothyroidism (CH) is not fully understood yet. Only genes currently known as defective in CH are reported.

**Figure 1:**
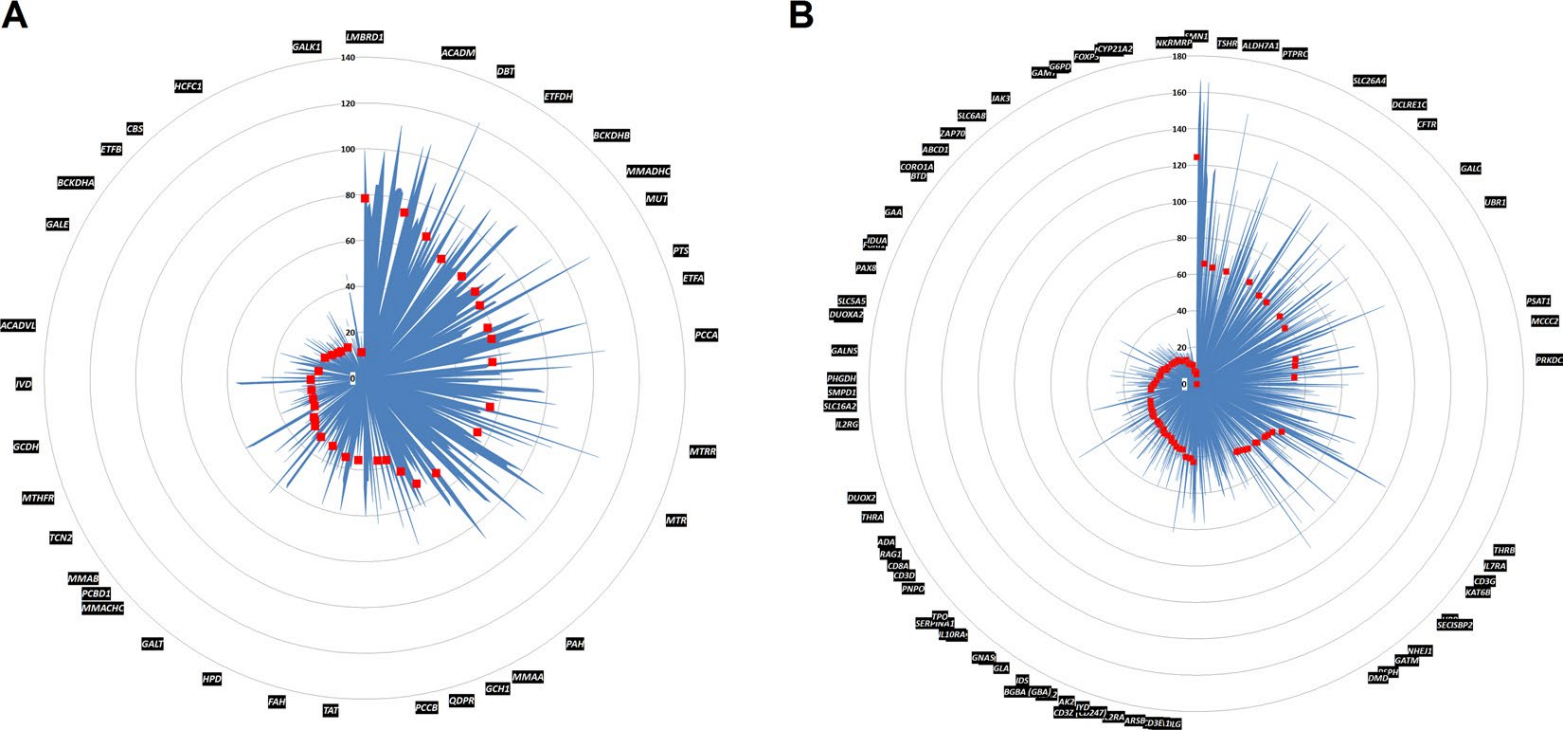
Mean depth of coverage for the different exons of selected genes. Blue shape represents the mean coverage for each exon. Red markers represent the mean coverage by a gene; these markers are sorted in decreasing order. (**A**) The 35 IEM genes included in the NBS program of the FWB. (**B**) Additional disorders that either are considered by different NBS initiatives worldwide or could benefit from early preventive care.

Coverage of the different exons for each gene highly fluctuated; some regions were uncovered, while other regions had a read depth of up to 238-fold. The number of reads for the different detected mutations varied between 8 and 83x. This coverage heterogeneity among the different selected genes is depicted in Fig. 1.

Nevertheless, all covered pathogenic mutations, either homozygote or compound heterozygote, for each patient have been identified by WES on DNA extracted from DBS. For patient DBS-14, MSUD was suspected initially upon newborn screening based on leucine/isoleucine levels (1262 µmol/L). Subsequent amino acid analysis identified the pathognomonic presence of allo-isoleucine, thus confirming the disorder. As molecular testing had not yet been requested, mutations had not been previously characterized by Sanger sequencing. We intended then to identify the pathogenic defects in *DBT*, *BCKDHA*, or *BCKDHB*. Unfortunately, the diagnosis of MSUD could not be confirmed based on coding sequence analysis of the respective genes, although a new unreferenced heterozygote mutation, c.G742T (p.A248S), was identified in *BCKDHB*. Since a significant percentage of *DBT* pathogenic variants are deletions (both large and small)^17^, we cannot exclude a large deletion in this gene, even though the gene coverage for this patient was not significantly different from the other 14 samples analyzed. Thus, any causal intronic mutation cannot be ruled out. For patient DBS-12, the 4 base-pair deletion located in the *GALT* promoter region and associated with the Duarte 2 (D2) allele was not covered by the exome sequencing probes and thus could not be identified. Nonetheless, the other four mutations associated with the D2 haplotype have been correctly characterized. Determination of the 15 base-pair duplication in *MMAB* (patient DBS-5) was also critical, as it was neither annotated by Annovar^18^ nor automatically identified with IGV software. Only an explicit visualization of the region of interest in IGV allowed the insertion to be identified.

We also studied the “presumed benign” polymorphisms using Cartagenia Bench Lab CNV software (Leuven, Belgium). The putative clinical impact of these variants, evaluated with two prediction databases^19,20^, revealed some unexpected information (Table 3). For patient DBS-6 with Tyrosinemia type I, the homozygote mutation c.554-1G > T in *FAH* was easily confirmed, and the pathogenic nonsense homozygote c.2056C > T (p.Gln686Ter) mutation in *DUOX2* (read depth of 6x), known to cause thyroid dyshormonogenesis type 6 and congenital hypothyroidism^21^, was also identified. However, this 24-year-old patient presents fully normal thyroid function, with repeated normal thyroxin and thyrotropin values measured over several years. The sequencing data were confirmed on a separate NGS experiment (from DNA extraction to sequence interpretation) with better coverage (read depth of 27x), as well as by Sanger sequencing. Such a genotype/phenotype discrepancy is quite surprising for a premature termination variant, but the mutation is located downstream of the thyroperoxidase active site of the protein^22^; thus, we could not exclude a residual functional activity. Moreover, variant databases describe this mutation as pathogenic on the basis of a unique publication reporting a single patient who was heterozygous for the anomaly^21^. To our knowledge, no functional studies have ever been performed to determine the activity of the truncated protein. Therefore, our data indicate that this variant should be classified as variant of unknown significance.

**Table 3:** Number of variants annotated in the different samples (focused on the 109 genes considered), and the corresponding clinical relevance of filtered polymorphisms evaluated among different databases (MutationTaster and ClinVar).

	**Variants**	**Filtered** ^**a**^ **variants**	**MutationTaster**				**ClinVar**			
			**Benign**	**VUS** ^**b**^	**Pathogenic**	**Unknown**	**Benign**	**VUS** ^**b**^	**Pathogenic**	**Unknown**
DBS-1	343	13	0	4	1	8	5	2	2	4
DBS-2	318	22	1	11	1	9	8	2	1	11
DBS-3	347	12	0	6	0	6	4	4	1	3
DBS-4	474	25	0	13	0	12	11	3	1	10
DBS-5	366	12	0	4	1	7	4	0	1	7
DBS-6	366	13	0	6	1	6	6	1	2^c^	4
DBS-7	351	15	0	5	2	8	5	1	4	5
DBS-8	367	16	1	10	1	4	8	2	2	4
DBS-9	475	18	1	8	0	9	9	0	1	8
DBS-10	361	22	1	9	0	12	9	1	3	9
DBS-11	328	13	0	6	0	7	4	2	0	7
DBS-12	376	9	0	3	1	5	4	1	1	3
DBS-13	354	13	1	4	0	8	7	2	1	3
DBS-14	355	11	1	6	0	4	7	2	0	2
DBS-15	357	12	0	6	0	6	5	2	1	4

^a^Filtering criteria: frequency <1%, located in exon or splicing site (within the first 8 intronic nucleotides), non-synonymous.

^b^Variant of unknown significance.

^c^2056C > T nonsense homozygote mutation was identified in DUOX2 gene of patient DBS-6.

## Discussion

This pilot study demonstrates that the amount and the quality of DNA extracted from DBS are adequate to identify pathogenic mutations by high-throughput sequencing. Although samples and genes carrying mutations are in limited numbers and extrapolation of the results to larger cohorts should be done with some circumspection, our present report underlines some of the challenges that WES faces. Indeed, WES reveals the vast depth of fluctuations in coverage between regions, which could subsequently generate difficulties in interpreting variants. Copy number variations (CNVs) should also be detected with caution as the unambiguous identification of small or large allelic deletions by NGS can be challenging when coverage is poor. Moreover, as observed with the 15-base-pair duplication in *MMAB*, small CNVs are not easily identified by bioinformatics tools. Hopefully, with the next evolution towards whole-genome sequencing (WGS), several drawbacks of WES could be solved. Indeed, WGS offers better coverage uniformity and provides more reliable sequences. WGS also improves CNV identification without the need for target amplification and allows the identification of non-coding alterations^23^.

Expecting drastic cost reductions and process automation in the near future, we could easily imagine our experiments contributing to paving the way for “next-generation” neonatal screening programs, provided that new paradigms (clinical, political, economic, societal and ethical) are defined. The first revolution already occurred in the world of newborn screening approximately fifteen years ago with the implementation of tandem mass spectrometry^24^. Currently, while this technological progress continues to challenge enacted codes (i.e., the Wilson and Jungner criteria)^25–27^, the second revolution is underway. NGS is now positioned as a universal approach allowing the identification of many disorders with one technology. Considering that and the results of our pilot study, we aim to further assess the utility of massive sequencing in a larger population. Several technical and clinical aspects of this ambitious pursuit are discussed here.

Presently, high-throughput sequencing is laborious and does not meet the requirements of NBS programs. Very large amounts of useless data are generated, and consequently, the treatment of bioinformatics data and review of variants generate unacceptable turnaround times compared to those of current biochemical assays. The interest in using WES (or WGS) to replace targeted approaches has already been discussed^28,29^, and based on actual available technologies and knowledge, the implementation of a selective approach appears to be the better choice. Such a panel analysis would be intended to improve coverage homogenization and to ensure a minimal read depth threshold between regions of interest. Bioinformatics analysis would thus be facilitated, and the costs of analysis would be reduced. Additionally, with the expected development of automated bioinformatics pipelines, a significant reduction in NGS analysis time can be envisaged in the future. In such targeted approaches, the list of targeted genes should obviously not be restrictive, since newborn screening programs are constantly evolving as new therapies are developed.

To date, the costs of massive sequencing remain disproportionate compared to those of mass spectrometry-based approaches. Therefore, implementation of NGS technologies into NBS could probably be first considered as a combined metabolomics-genomics approach, with the sequencing focusing only on capturing conditions without reliable biomarkers. Indeed, our experiments allowed for accurate sequencing with acceptable coverage of the coding regions of some treatable disorders for which identification is not reliable using mass spectrometry techniques (e.g., pyridoxine-dependent epilepsy, cerebral creatine deficiency syndrome). Using sequencing only curable diseases that lack defined biomarkers would be intended to initially limit the costs of implementing NGS in NBS. Afterward, greatly increasing the number of samples tested using molecular techniques would help to reduce reagents and bioinformatics costs, subsequently supporting the sustainability of molecular NBS.

Applying WES (or WGS) to newborn screening may also present substantial benefits. Assuming that blood samples could be collected earlier (i.e., at the day of birth, eventually from cord blood), the medical care needs of affected neonates could be anticipated. Moreover, given the wide variability of screened disorders worldwide, harmonization of NBS programs could be facilitated with the implementation of such universal technologies. The acquisition of genomic sequences at birth may also be beneficial for individuals who become sick later in life. Indeed, presuming lifelong data storage on a secured and controlled server, retrospective consultations of patients data could be helpful to reduce delays in the diagnosis of rare diseases^30^. Access to patient’s information in such instances should obviously be driven by strict clinical and ethical constraints.

Careful consideration will also need to be given to unexpected and medically irrelevant incidental findings. As reported for patient DBS-6, an unexpected homozygote variant that was previously considered a pathogenic has been characterized in a gene unrelated to the patient’s disorder, questioning the reliability of some variants referenced in databases. Heterozygous carriers of recessive defects are characterized unequivocally, and polymorphisms and intermediate deficiencies requiring no intervention are also identified. These results might burden medical practices (increasing unnecessary documentation as well as anxiety in healthy carriers) and possibly cripple healthcare budgets. Substantial efforts will thus be needed to clarify genotype/phenotype correlations, and large studies are required to associate unequivocal biochemical defects with gene variants. Our knowledge of the genome will subsequently be improved and will progressively enhance the sensitivity and specificity of these assays.

With these new high-throughput technologies, the current restriction focusing the screening to diseases for which effective treatment is available could also be reconsidered. This limitation confines, among other things, the clinical trials to symptomatic patients and ignores the potential benefits of any preventive intervention. Early identification of patients for other conditions could probably allow pre-symptomatic therapies in randomized studies. Additionally, the feasibility of the voluntary expansion of screening, providing the choice to families who want to know about other conditions, is already under debate^31–34^. Educational challenges in the training of health professionals and in information provided to the public should also be considered. Parents should be informed of the screening perimeter, its implications and the follow-up required. Appropriate infrastructure should ensure care, education and follow-up. Specific registries should be set up to provide the opportunity for families to include children in clinical trials for new treatments.

Finally, the emergence of the NGS era will call into question the current neonatal screening dogma. Old doctrines should not be barriers to the emergence of new expectations: scientific and technological advances must obviously be encouraged, but they cannot be made without any clinical, political, economic, societal and ethical debates^35,36^. Accordingly, the National Human Genome Research Institute already promotes an Ethical, Legal, and Social Implications (ELSI) Program to anticipate and address these issues^37^.

## Methods

### Samples

Fifteen patients with confirmed IEM were considered in this study. Almost all patients were identified by newborn screening, and for all except one, mutations were initially characterized by Sanger sequencing during diagnostic work-up.

In the course of the patient’s clinical follow-up, amino acid or acylcarnitine profiles are routinely analyzed, and for logistical considerations, whole blood is collected on filter paper. Ethical approval (reference B707201421546) was obtained from the Institutional Review Board (Ethical Committee of the Faculty of Medicine of the University of Liege), in compliance with the Declaration of Helsinki. All experiments were performed in accordance with relevant guidelines and regulations, and all patients or their legal representatives signed a written informed consent form. This work consisted of a prospective study and did not lead to any changes in the treatment of enrolled patients. Only residual DBS were used to perform exome sequencing.

### DNA Extraction

Experiments were performed using five blood spots (3.1 mm diameter) for each patient. DNA was extracted from DBS according to the protocol recently published by St Julien and collaborators^38^, with slight modifications. The amounts of DNA were estimated, and the quality of the retrieved material was assessed using the KAPA hgDNA Quantification and QC^®^ kit (Kapa Biosystems), which is designed to amplify targets of 41 base pairs (bp), 129 bp, and 305 bp within a conserved single-copy locus in the human genome. Absolute quantification is achieved using the 41 bp assay, while the longer amplicons are used to assess DNA quality. Since DNA damage has a greater impact on the amplification of longer targets, the relative quality of a DNA sample can be inferred by normalizing the concentration obtained using the 129 bp or 305 bp assay against the concentration obtained with the 41 bp assay. This normalization generates “Q-ratios” with values between 0 and 1, which can be used as a relative measure of DNA quality prior to NGS library construction.

### Sequencing

**Figure 2:**
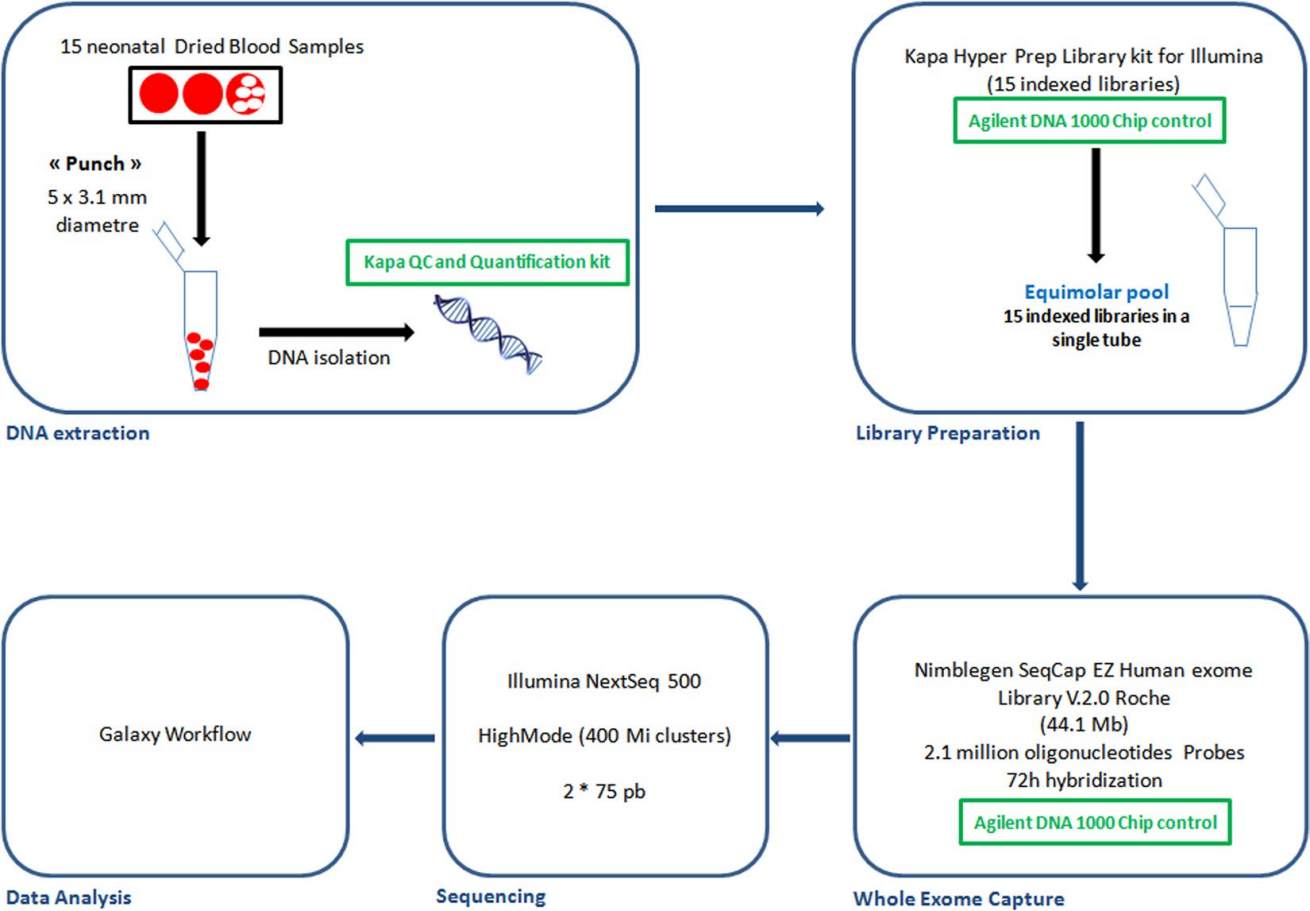
Overview of analytical workflow.

Briefly, 100 ng of extracted DNA was fragmented (Bioruptor^®^, Diagenode) and used to prepare indexed libraries (SeqCap EZ Indexed Adapters; Roche) with the KAPA Hyper Prep^®^ kit (Kapa Biosystems). These libraries were pooled equimolarly and incubated with probes to capture all coding exons (44.1 Mb target) (SeqCap EZ Human Exome library v.2.0; Roche). Sequencing was performed with 2*75 bp reads on a high mode NextSeq. 500 run. The entire analytical process is illustrated in Fig. 2.

### Data Processing

**Figure 3:**
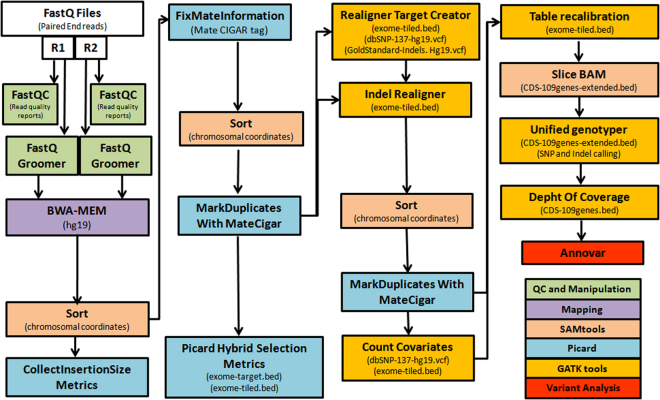
Framework for variation discovery and genotyping from NGS sequencing.

A bioinformatics flowchart is presented in Fig. 3 ^39^. Data analysis was performed using Galaxy tools on the usegalaxy.org server^40^. Raw reads were mapped against a reference genome (GRCh37/hg19) with BWA-MEM version 0.7.15.1. PCR duplicates were flagged with Picard version 2.7.1. Indel realignment, base quality recalibration and coverage depth calculations were optimized with GATK version 3.8. Sequences were visualized with IGV (Integrative Genomics Viewer)^41^. Anonymized data were stored under controlled access on a secured server.

## References

[1] Beale S, Sanderson D, Sanniti A, Dundar Y, Boland A (2015). A scoping study to explore the cost-effectiveness of next-generation sequencing compared with traditional genetic testing for the diagnosis of learning disabilities in children. Heal. Technol Assess.

[2] Tang W (2009). DNA extraction from formalin-fixed, paraffin-embedded tissue. Cold Spring Harb Protoc.

[3] Butler, J. M. The future of forensic DNAanalysis. *Philos Trans R Soc L. B Biol Sc*i **370**, (2015). 10.1098/rstb.2014.0252 PMC4580997 26101278

[4] CLSI. Blood Collection on Filter Paper for Newborn Screening Programs; Approved Standard—Sixth Edition. *Clin. Lab. Stand. Inst. Doc*. **33**, (2013).

[5] Schrynemackers-Pitance P, Schoos-Barbette S (1987). Determination of aromatic and neutral aminoacids by HPLC in blood specimens collected on filter paper. Clin Chim Acta.

[6] Shokati, T. *et al*. Quantification of the Immunosuppressant Tacrolimus on Dried Blood Spots Using LC-MS/MS. *J Vis Exp*10.3791/52424 (2015). 10.3791/52424 PMC4692696 26575262

[7] Tretzel L (2014). Use of dried blood spots in doping control analysis of anabolic steroid esters. J Pharm Biomed Anal.

[8] Napierala Mavedzenge S (2015). Finger Prick Dried Blood Spots for HIV Viral Load Measurement in Field Conditions in Zimbabwe. PLoS One.

[9] Barben J (2012). Retrospective analysis of stored dried blood spots from children with cystic fibrosis and matched controls to assess the performance of a proposed newborn screening protocol in Switzerland. J Cyst Fibros.

[10] Hollegaard MV (2013). Archived neonatal dried blood spot samples can be used for accurate whole genome and exome-targeted next-generation sequencing. Mol Genet Metab.

[11] Cantarel BL (2015). Analysis of archived residual newborn screening blood spots after whole genome amplification. BMC Genomics.

[12] Poulsen JB (2016). High-Quality Exome Sequencing of Whole-Genome Amplified Neonatal Dried Blood Spot DNA. PLoS One.

[13] Burgard P (2012). Newborn screening programmes in Europe; arguments and efforts regarding harmonization. Part 2 - From screening laboratory results to treatment, follow-up and quality assurance. in. Journal of Inherited Metabolic Disease.

[14] Loeber JG (2012). Newborn screening programmes in Europe; arguments and efforts regarding harmonization. Part 1 - From blood spot to screening result. in. Journal of Inherited Metabolic Disease.

[15] Moat SJ, Bradley DM, Salmon R, Clarke A, Hartley L (2013). Newborn bloodspot screening for Duchenne muscular dystrophy: 21 years experience in Wales (UK). Eur. J. Hum. Genet..

[16] Phan HC, Taylor JL, Hannon H, Howell R (2015). Newborn screening for spinal muscular atrophy: Anticipating an imminent need. Semin. Perinatol..

[17] Strauss, K., Puffenberger, E. & Morton, D. *Maple Syrup Urine Disease*. (GeneReviews® [Internet]. University of Washington, 2013).

[18] Yang H, Wang K (2015). Genomic variant annotation and prioritization with ANNOVAR and wANNOVAR. Nat Protoc.

[19] NCBI Resource Coordinators. Database resources of the National Center for Biotechnology Information. *Nucleic Acids Res*. **44**, D7–D19 (2015). 10.1093/nar/gkv1290 PMC4702911 26615191

[20] Schwarz JM, Rödelsperger C, Schuelke M, Seelow D (2010). MutationTaster evaluates disease-causing potential of sequence alterations. Nat. Methods.

[21] Moreno JC (2002). Inactivating Mutations in the Gene for Thyroid Oxidase 2 (*THOX2*) and Congenital Hypothyroidism. N. Engl. J. Med..

[22] Grasberger H (2010). Defects of thyroidal hydrogen peroxide generation in congenital hypothyroidism. Molecular and Cellular Endocrinology.

[23] Meienberg J, Bruggmann R, Oexle K, Matyas G (2016). Clinical sequencing: is WGS the better WES?. Hum. Genet..

[24] Chace DH, Kalas TA, Naylor EW (2002). The application of tandem mass spectrometry to neonatal screening for inherited disorders of intermediary metabolism. Annu Rev Genomics Hum Genet.

[25] Kronn D, Mofidi S, Braverman N, Harris K (2010). Diagnostic guidelines for newborns who screen positive in newborn screening. Genet Med.

[26] Ombrone D, Giocaliere E, Forni G, Malvagia S, la Marca G (2016). Expanded newborn screening by mass spectrometry: New tests, future perspectives. Mass Spectrom Rev.

[27] Wilson, J. M. G. & Jungner, G. *Principles and practice of screening for disease*. (WHO, 1968).

[28] Francescatto L, Katsanis N (2015). Newborn screening and the era of medical genomics. Semin Perinatol.

[29] Qian, J. *et al*. Applying targeted next generation sequencing to dried blood spot specimens from suspicious cases identified by tandem mass spectrometry-based newborn screening. *J. Pediatr. Endocrinol. Metab*. 10.1515/jpem-2017-0003 (2017). 10.1515/jpem-2017-0003 28771436

[30] Zurynski Y (2017). Australian children living with rare diseases: experiences of diagnosis and perceived consequences of diagnostic delays. Orphanet J. Rare Dis..

[31] Bailey DB, Gehtland L (2015). Newborn screening: evolving challenges in an era of rapid discovery. JAMA.

[32] Lewis MA (2016). Supporting Parental Decisions About Genomic Sequencing for Newborn Screening: The NC NEXUS Decision Aid. Pediatrics.

[33] Howard HC (2015). Whole-genome sequencing in newborn screening? A statement on the continued importance of targeted approaches in newborn screening programmes. Eur J Hum Genet.

[34] Almannai M, Marom R, Sutton VR (2016). Newborn screening: a review of history, recent advancements, and future perspectives in the era of next generation sequencing. Curr Opin Pediatr.

[35] Knoppers BM, Senecal K, Borry P, Avard D (2014). Whole-genome sequencing in newborn screening programs. Sci Transl Med.

[36] Botkin JR, Rothwell E (2016). Whole Genome Sequencing and Newborn Screening. Curr Genet Med Rep.

[37] McEwen JE (2014). The Ethical, Legal, and Social Implications Program of the National Human Genome Research Institute: reflections on an ongoing experiment. Annu Rev Genomics Hum Genet.

[38] St Julien KR (2013). High quality genome-wide genotyping from archived dried blood spots without DNA amplification. PLoS One.

[39] DePristo MA (2011). A framework for variation discovery and genotyping using next-generation DNA sequencing data. Nat. Genet..

[40] Afgan E (2016). The Galaxy platform for accessible, reproducible and collaborative biomedical analyses: 2016 update. Nucleic Acids Res.

[41] Thorvaldsdottir H, Robinson JT, Mesirov JP (2013). Integrative Genomics Viewer (IGV): high-performance genomics data visualization and exploration. Br. Bioinform.

